# Transcriptome Analysis Reveals Cotton (*Gossypium hirsutum*) Genes That Are Differentially Expressed in Cadmium Stress Tolerance

**DOI:** 10.3390/ijms20061479

**Published:** 2019-03-24

**Authors:** Mingge Han, Xuke Lu, John Yu, Xiugui Chen, Xiaoge Wang, Waqar Afzal Malik, Junjuan Wang, Delong Wang, Shuai Wang, Lixue Guo, Chao Chen, Ruifeng Cui, Xiaoming Yang, Wuwei Ye

**Affiliations:** 1 Institute of Cotton Research of Chinese Academy of Agricultural Science, State Key Laboratory of Cotton Biology, Key Laboratory for Cotton Genetic Improvement, Anyang 455000, Henan, China; h13707663917@163.com (M.H.); 15824990556@163.com (X.L.); pycxg-007@163.com (X.C.); wangxiaoge1990@126.com (X.W.); Waqarviqi244@gmail.com (W.A.M.); Wjj2004liyuan@sina.com (J.W.); wdl_21@126.com (D.W.); wangshuai_19871201@163.com (S.W.); guolixue0114@163.com (L.G.); cc1218@163.com (C.C.); xiaocui0126@126.com (R.C.); xmyang152@163.com (X.Y.); 2 USDA-ARS Southern Plains Agricultural Research Center, College Station, TX 77845, USA; john.yu@ars.usda.gov

**Keywords:** cotton (*Gossypium hirsutum* L.), transcriptome, Cd stress, *GhHMAD5*, overexpression, VIGS (virus induced gene silence)

## Abstract

High concentrations of heavy metals in the soil should be removed for environmental safety. Cadmium (Cd) is a heavy metal that pollutes the soil when its concentration exceeds 3.4 mg/kg. Although the potential use of cotton to remediate heavy Cd-polluted soils is known, little is understood about the molecular mechanisms of Cd tolerance. In this study, transcriptome analysis was used to identify Cd tolerance genes and their potential mechanisms in cotton. We exposed cotton plants to excess Cd and identified 4627 differentially expressed genes (DEGs) in the root, 3022 DEGs in the stem and 3854 DEGs in the leaves through RNA-Seq analysis. Among these genes were heavy metal transporter coding genes (ABC, CDF, HMA, etc.), annexin genes and heat shock genes (HSP), amongst others. Gene ontology (GO) analysis showed that the DEGs were mainly involved in the oxidation–reduction process and metal ion binding. The DEGs were mainly enriched in two pathways, the influenza A and pyruvate pathway. GhHMAD5, a protein containing a heavy-metal binding domain, was identified in the pathway to transport or to detoxify heavy metal ions. We constructed a *GhHMAD5* overexpression system in *Arabidopsis thaliana* that showed longer roots compared to control plants. GhHMAD5-silenced cotton plants showed more sensitivity to Cd stress. The results indicate that *GhHMAD5* is involved in Cd tolerance, which gives a preliminary understanding of the Cd tolerance mechanism in upland cotton. Overall, this study provides valuable information for the use of cotton to remediate soils polluted with Cd and potentially other heavy metals.

## 1. Introduction

Cadmium (Cd), one of the most common heavy metals with the strongest toxicity (exceeding 3.4 mg/kg in soil) [1], causes significant pollution to farmland, and actively transfers in the soil–plant system [2]. If the concentration of Cd in the plant reaches a certain level, toxic symptoms appear, such as crinkled and turned yellowing of plant leaves, degraded chloroplasts, closed stomata, and an imbalance of moisture metabolism, and by inhibiting the functional enzyme activity, it facilitates the decomposition of ascorbic acid and damages the chlorophyll. These factors can lead to a decline in crop yield and quality. The Cd absorbed by the plant will enter into the food chain, affecting the metabolism of calcium and phosphorus in the human body, and even increasing the possibility of teratogenicity and cancer [3]. Thus elimination of Cd from the soil and reduction of its harmful effects on human life, are of great importance.

The traditional methods to counteract heavy metal pollution are physical and chemical, but these are time-consuming and laborious, and can cause secondary pollution (i.e., primary pollutants under physical or chemical conditions result in new pollutants) [4]. At present, phytoremediation, a novel strategy for the removal of heavy metals from the soil using plants, has been introduced to resolve Cd pollution. A large number of plants were identified as able to remove Cd but had small biomass and slow growth, and therefore cannot be widely used to treat contaminated soil [5]. The most effective way for remediation of Cd polluted soil is to cultivate plants that can accumulate the maximum amount of Cd in their specific organs, which will not only eliminate Cd from the soil, but also maintain the rational use of the land to achieve sustainable production.

Cotton, one of the major economic crops grown all over the world has a large planted area of about 3.39 × 10^7^ hectares worldwide [6]. It is reported that cotton, strongly tolerant of Cd, can be used as a restoration plant for Cd-contaminated soil [7]. It is widely believed that cotton is suitable for planting in industrially polluted areas [8]. Daud et al. found that the treatment of cotton seeds with a low concentration of Cd (10–100 μm) significantly increased their germination rate, which decreased with 1 mM concentration of Cd [9]. The activity of SOD, APX and other enzymes decreased in cotton under Cd stress [10]. Lawali et al. found significant differences in the toxicity of Cd in different cotton varieties [11]. The order of absorption of Cd in all parts of the reproductive organs of cotton was kernel > bell shell > fiber, with a significant difference among varieties. Cotton was reported as a remediation crop in heavy metal contaminated areas with a resilient absorptive capacity for heavy metals and tolerance against Cd stress [12], and with few effects on cotton fibre quality [13]. The previous studies of Cd stress in cotton mainly concentrated on physiological and biochemical aspects. We believe it is of great significance to uncover the Cd tolerance genes in cotton, and to analyze the regulation network of cotton Cd tolerance for the remediation of Cd-contaminated soil by molecular technology.

RNA-Seq can be adopted to analyze gene expression of plants under various biotic and abiotic stresses [14,15], and has been successfully used under Cd stress on many plants, such as ramie [16], maize [17], and rice [18]. Previous research has also reported a lot of proteins and gene families related to Cd stress, such as ABC transporters, Nramp family proteins, Zinc finger transporter families, YSL family proteins [19,20], P-type ATPase family proteins [21], and the CDF families [22].

Cd has been reported to enter organs through the cytomembrane via the Ca^2+^ pathway [23], inducing many free radicals and reactive oxygen species (ROS) to produce oxidative stress [4,24]. By adding Ca^2+^ or Mg^2+^ ion into the Cd stress solution, the degree of damage to soybean from Cd can be alleviated [25]. The Ca^2+^ signal transduction pathway plays a key role under Cd stress [26]. Overexpression of *SpHMA3* in *Sedum alfredii* could enhance Cd tolerance [27]. Previous studies indicated that there was a close relationship between the transport of glutathione reductase (GR) and the accumulation of jasmonic acid in *Lycium barbarum* under Cd stress [28]. The ascorbic acid (ASA) and glutathione (GSH) in the leaf significantly reduced in maize seedlings under Cd stress [29]. Three bHLH transcription factors (*FIT, AtbHLH38,* and *AtbHLH39*) of *Arabidopsis* were reported to be involved in the plant’s response to Cd stress; the transgenic plants were more tolerant to Cd than the wild type plants [30]. In this study, DEGs were explored and the regulation network under Cd stress was constructed by cotton transcriptome sequencing, and the gene function of *GhHMAD5* was validated. This study would provide more information for understanding the mechanism of tolerance to Cd in cotton, and lay the foundation to repair heavy metal contaminated soil through molecular breeding methods.

## 2. Results

### 2.1. Phenotypic Analysis of Cotton under Cd Stress

**Figure 1:**
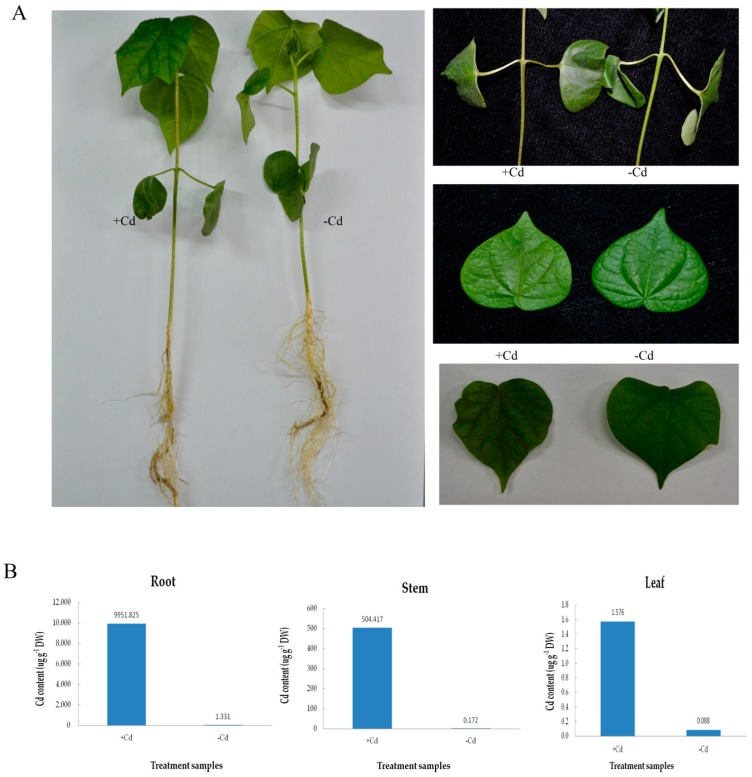
Phenotypic symptoms (**A**) and Cd content (**B**) in roots, stems and leaves of Han242 cotton seedlings with or without 4 mM Cd stress for 9 h.

Han 242, cotton cultivar (*Gossypium hirsutum* L.), was treated with 4 mM CdCl_2_ compared with the control, which was subjected to the same amount of pure water. Phenotypic characters on cotton roots, stems, and leaves are shown in Figure 1A. The stems turned black, the leaves turned yellow and the veins lost their green pigment in Cd-treated plants compared to the controls. Over time, the cotton leaves became dry, and the stems turned black. The phenotypic traits of different cotton varieties was shown (Figure S1) under the same Cd concentration stress. Under Cd stress, remarkable accumulation of Cd was found in their root, stem and leaf tissues after 9 h. The results also indicated that the root and leaf tissues showed the highest and the lowest Cd content, respectively, compared to the stem (Figure 1B). The Cd content of the cotton also indicated that cotton has a strong absorptive capacity for Cd.

### 2.2. Quality Analysis of the Transcriptome Sequence

**Figure 2:**
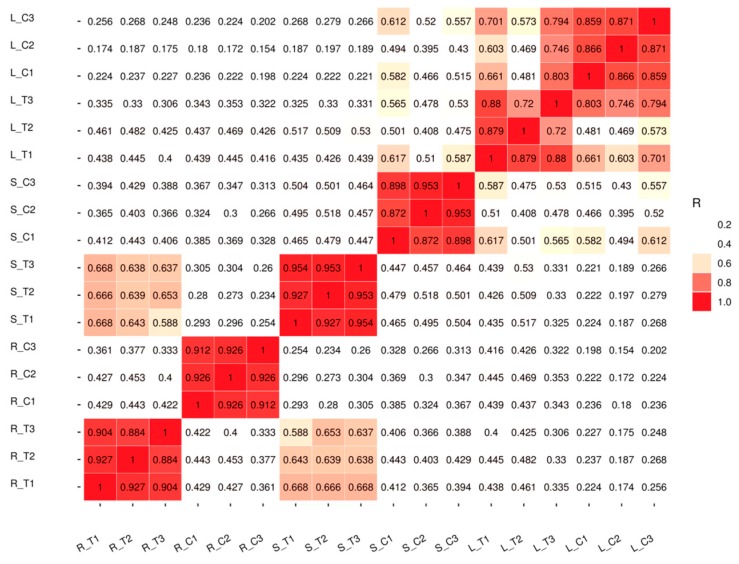
Pearson correlation between samples. R_T1: Root Cd treatment 1, R_T2: Root Cd Table 2. R_T3: Root Cd treatment 3, R_C1: Root water control 1, R_C2: Root water control 2, R_C3: Root water control 3, S_T1: Stem Cd treatment 1, S_T2: Stem Cd treatment 2, S_T3: Stem Cd treatment 3, S_C1: Stem water control 1, S_C2: Stem water control 2, S_C3: Stem water control 3, L_T1: Leaf Cd treatment 1, L_T2: Leaf Cd treatment 2, L_T3: Leaf Cd treatment 3, L_C1: Leaf water control 1, L_C2: Leaf water control 2, L_C3: Leaf water control 3. The colors of the box represent the degree of correlation; red color represents the highest degree of correlation and white color represents the lowest degree of correlation.

The samples of roots, stems, and leaves of treated plants and controls were collected separately, and three biological replicates were conducted for both groups (treated versus control). Totally 18 qualified libraries were established. The raw reads were filtered and low quality reads were removed to get clean reads. Approximately 909 million clean valid reads were done, which contained 136.49 Gb of sequence data. Over 97.77% of the clean reads at a Q20 level and over 89.02% of the clean reads at a Q30 level were obtained. The GC content of the sequence data reached 44% (Table 1). Pearson correlation coefficient (PCC) analysis was performed on 18 established libraries to check the correlation between several tissues of all the samples (Figure 2). A dendrogram of raw RNA-Seq reads from all samples was acquired (Figure S7). A close correlation in root tissue was observed between the treated and control groups. The reason may be because of tissue specificity or root tissue being the first tissue to respond to Cd stress. Principal component analysis (PCA) showed that the repeatability was satisfactoryaccordance among the three samples (Figure S5). Above all, the results of transcriptome sequencing were reliable. Purified and valid reads were mapped into the *G.hirsutum* reference genome from CottonGen with a high proportion rate (Figure S6). The raw sequence data of RNA-Seq were submitted to NCBI with an accession number of GSE126671.

### 2.3. Analysis of Differential Expression Genes under Cd Stress in Cotton

Comparative analysis between the controls and the treated samples to check the transcriptional changes in response to Cd stress was conducted using *cuffdiff* software. Gene expression profiles were comprised of 135,162 genes that consisted of 117,377 annotated genes and 17,785 novel genes (Table S5).

**Figure 3:**
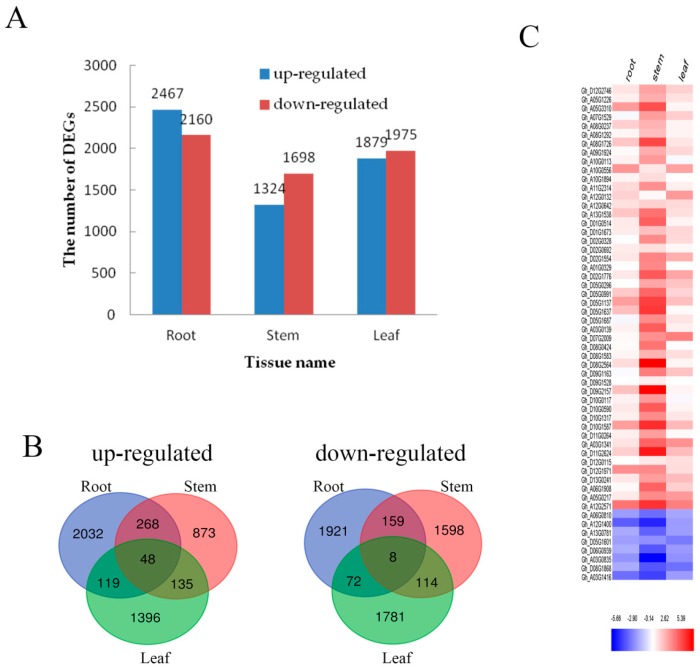
DEGs in roots, stems and leaves. (**A**) The number of genes up- or down-regulated by *fold change* ≥ 2 (*p* < 0.05) in roots, stems and leaves under Cd stress. (**B**) Venn diagrams showing the unique and shared regulated genes in cotton roots, stems and leaves under Cd stress; (**C**) The relative expression analysis of 56 genes that are expressed in roots, stems and leaves. The fold-change ratios of the genes are indicated by the different colors. The red color represents the highest expression, the blue color represents the lowest expression.

To effectively analyze and interpret the DEGs of the transcriptome, the *P-value* < 0.05 and |log_2_ *fold change*| ≥ 2 was used, and an overall distribution of the DEGs was visualized in every tissue (Figure 3A). Overall 4627 DEGs were found in the root section including 2467 up-regulated genes and 2160 down-regulated genes. Similarly, 3022 DEGs were found involved in stem portions that consisted of 1324 up-regulated and 1698 down-regulated genes, whereas a total of 3854 DEGs were observed in leaves including 1879 up-regulated genes and 1975 down-regulated genes. Furthermore, a Venn diagram was developed to show the statistical analysis of DEGs in each tissue (Figure 3B). For the up-regulated mechanism, it showed 316 DEGs in the root–stem group, 183 DEGs in the stem–leaf group and 167 DEGs in the root–leaf group. Whereas 167 down-regulated DEGs were found in the root–stem, 122 in the stem–leaf and 80 in the root–stem groups. Results indicated that there were 3953 DEGs (up-regulated: 2032; down-regulated: 1921) of roots, 2471 DEGs (up-regulated: 873; down-regulated: 1598) of stems and 3177 DEGs (up-regulated: 1396; down-regulated: 1781) of leaves under tissue specific expression.

Under Cd stress, 56 DEGs (up-regulated: 48; down-regulated: 8) were identified in the root, stem and leaf (Figure 3B). In order to analyze the expression intensity of these 56 DEGs in roots, stems and leaves, the result was illustrated by heat map (Figure 3C). A large proportion of these 56 DEGs were evidently expressed in the stem, which maintained its consistency with continued Cd stress. The number of DEGs in roots, stems and leaves was ranked as root > stem > leaf. This data supported the fact that the root responded first and strongly to Cd stress.

### 2.4. Expression of Heavy Metal Transport Proteins in Cotton under Cd Stress

**Figure 4:**
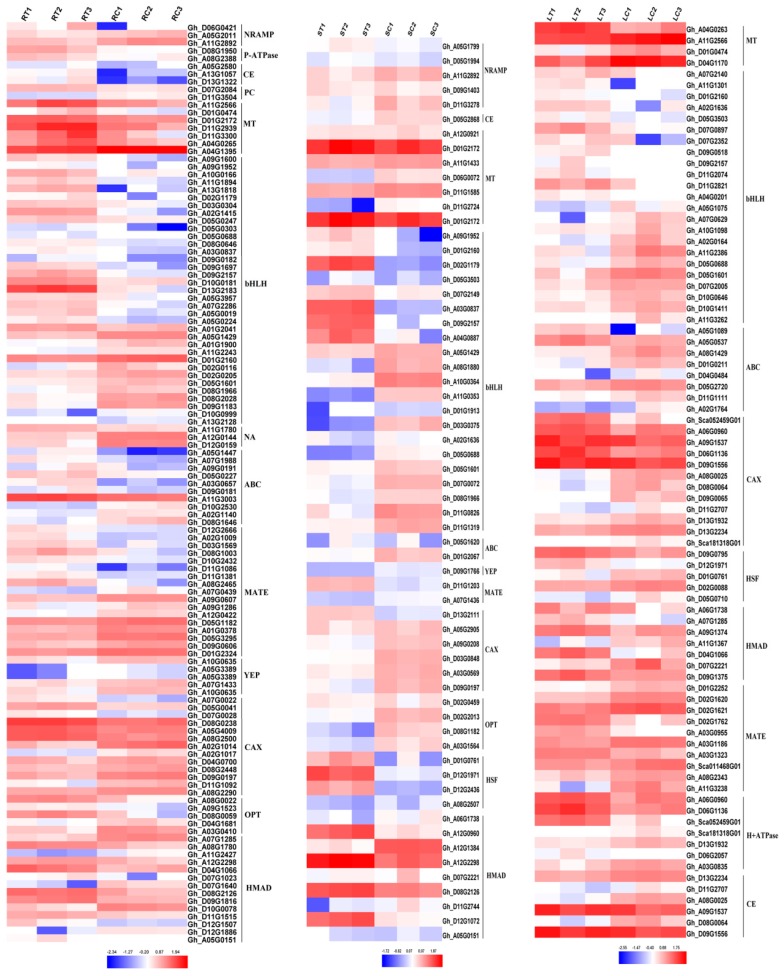
Metal transporters expressed in roots, stems and leaves under Cd stress. RT1: Root Cd treatment 1, RT2: Root Cd treatment 2, RT3: Root Cd treatment 3, RC1: Root water control 1, RC2: Root water control 2, RC3: Root water control 3, ST1: Stem Cd treatment 1, ST2: Stem Cd treatment 2, ST3: Stem Cd treatment 3, SC1: Stem water control 1, SC2: Stem water control 2, SC3: Stem water control 3, LT1: Leaf Cd treatment 1, LT2: Leaf Cd treatment 2, LT3: Leaf Cd treatment 3, LC1: Leaf water control 1, LC2: Leaf water control 2, LC3: Leaf water control 3. The fold-change ratios of the genes are indicated by the different colors. The red color represents the highest expression, the blue color represents the lowest expression. CE: cation efflux family, ABC: ATP-binding cassette transporter, CAX: calcium exchanger, NRAMP: natural resistance-associated macrophage protein, NA: nicotianamine, OPT: peptide transporter, PC: phytochelation, MT: metallothionein, MATE: multidrug resistance-associated protein, YEP: yellow stripe protein, bHLH: basic helix-loop-helix DNA-binding protein, HMAD: heavy metal transporter. HSF: heat shock factor.

Heavy metal transporters play a vital role in the plants metabolism under Cd Stress. There are a large number of transporters in cotton (Figure 4), such as Nramp, P-type ATPase, CE, PC, MT, bHLH, Zip, ABC, YSL, MATE, CAX, OPT, HSP and ferredoxin. The P-type ATPase and PC did not show a significant expression in the stems, while Nramp, P-type ATPase and PC also did not express in the leaves, which was due to tissue specificity. Overexpressed *NRAMP* gene has been reported to enhance Cd tolerance in *Arabidopsis thaliana* [31], and overexpressed *PC* and *MT* genes showed increased Cd tolerance in *Escherichia coli* and *Arabidopsis thaliana* [32,33].

### 2.5. Functional Classification of DEGs

The functional classification of DEGs were determined by using gene ontology (GO) terms based on their corresponding biological processes, cellular components, and molecular functions. Similar molecular biological processes and molecular functions were observed in cotton roots, stems and leaves under Cd stress.

**Figure 5:**
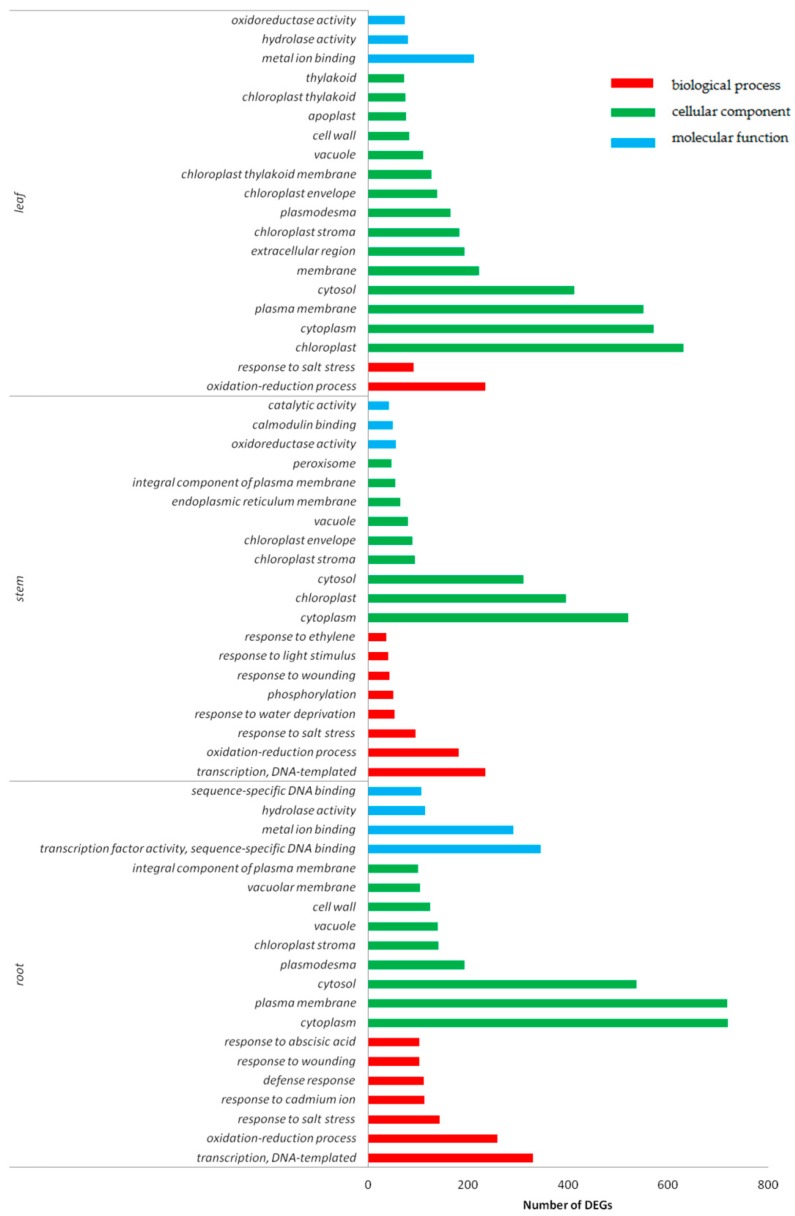
GO (gene ontology) function classification of DEGs in roots, stems, and leaves under Cd stress. Red color represents the biological process, green color represents cellular component, and blue color represents molecular function.

GO enrichment analysis was performed by *p*-value, and the top 20 functional terms with the minimum *p*-value and the most specific genes were selected for statistical analysis (Figure 5). It was found that the biological functions of the DEGs in root, stem and leaf tissues was enriched in the oxidation–reduction process responding to salt stress. In roots, the DEGs were found enriched in metal ion binding, hydrolase activity, and copper ion binding. Among the most abundant genes in stems were enriched in oxidoreductase activity, calmodulin binding, and pyridoxal phosphate binding. The DEGs of leaves were found most abundant in metal ion binding, oxidoreductase activity, and catalytic activity. The DEGs enriched in cellular components were found in the cell nucleus, cytoplast, and plasma membrane in cotton.

**Figure 6:**
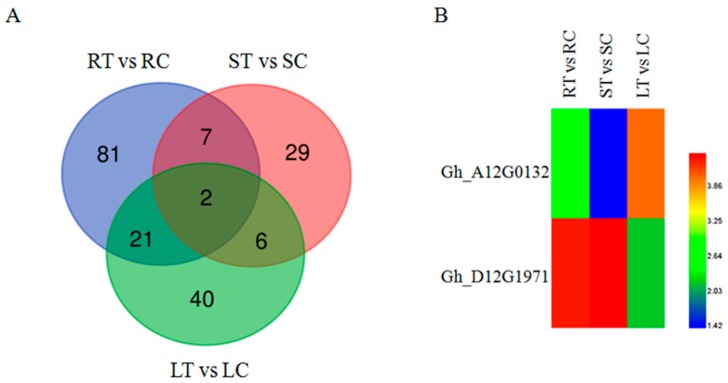
(**A**) Venn diagrams of DEGs in the GO term (GO: 0046686) under Cd stress; (**B**) Cluster map of DEGs in the GO term under Cd stress (GO: 0046686). The red color represents the highest expression, the blue color represents the lowest expression. The samples treated with CdCl_2_ were called RT, ST and LT. The samples not treated with CdCl_2_ were called RC, SC, and LC, respectively. Then ‘R’ indicates the root tissue, ‘S’ indicates the stem tissue, ‘L’ indicates the leaf tissue, ‘T’ indicates the treated, ‘C’ indicates the control.

One GO term (GO: 0046686) was classified as responding to Cd stress based on GO annotation analysis. Figure 6A shows the total number of genes responding to Cd stress in roots, stems and leaves by Venn diagram. There were 111 DEGs in the roots, 44 DEGs in the stems and 69 DEGs in the leaves, with 150 DEGs (root: 81, stem: 29, leaf: 40) under tissue specific expression. Among these 150 DEGs, 2 DEGs were identified as up-regulated genes in roots, stems, and leaves, which were Gh_A12G0132 (Aldolase-type TIM barrel family protein) and Gh_D12G1971 (mitochondrion-localized small heat shock protein 23.6).

### 2.6. KEGG Analysis of DEGs

**Figure 7:**
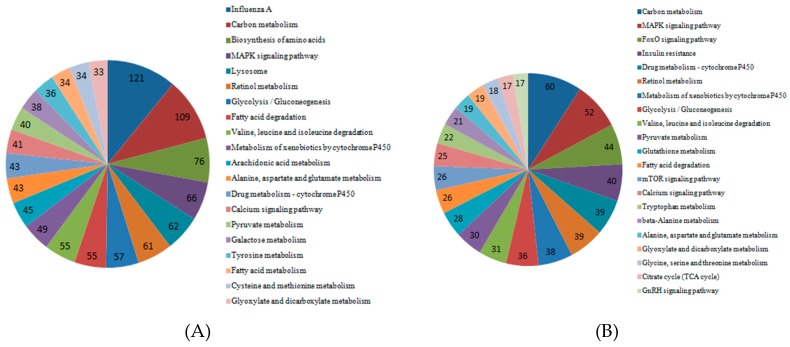

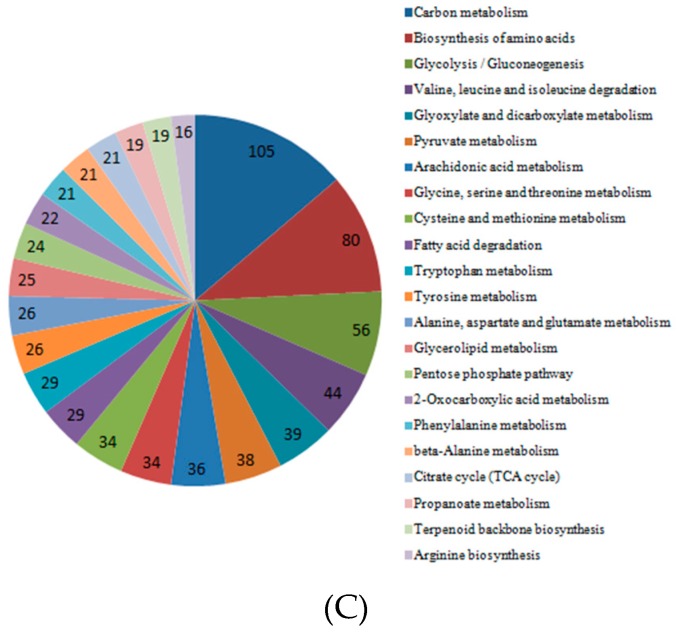
Significant pathways and numbers based on KEGG analysis of DEGs in cotton roots, stems, and leaves. (**A**) Significant pathways and numbers of DEGs in roots; (**B**) significant pathways and numbers of DEGs in stems; (**C**) significant pathways and numbers of DEGs in leaves.

To determine the pathway for DEGs under Cd stress, KOBAS was used for gene annotation. We obtained the enriched pathway map of DEGs in roots, stems, and leaves (Figure 7). The graph showed that DEGs in the roots played a role in influenza A, carbon metabolism, and biosynthesis of amino acids. In KEGG pathway analysis, DEGs enriched in the stems were important in carbon metabolism, the MAPK signaling pathway and drug metabolism–cytochrome P450. The DEGs enriched in the leaves played a role in carbon metabolism, biosynthesis of amino acids, and glycolysis/gluconeogenesis.

### 2.7. Expression of Transcription Factors under Cd Stress of Cotton

It was reported that transcription factors played an important role in response to stresses [34]. To understand the behavior of transcription factors in cotton in response to Cd stress, we analyzed transcription factors of different genes in the roots, stems, and leaves (Figure S2). Many transcriptional factors expressed under Cd stress have been reported in previous studies, such as NAC, bHLH, WRKY, etc [35,36,37]. In this study, many unreported transcriptional factors were discovered, such as C3H, C2H2, Orphans, and MYB, etc, whereas C2H2 has been previously reported associated with osmotic stress in *Arabidopsis thaliana* [38].

### 2.8. Verification of Sequence Data

**Figure 8:**
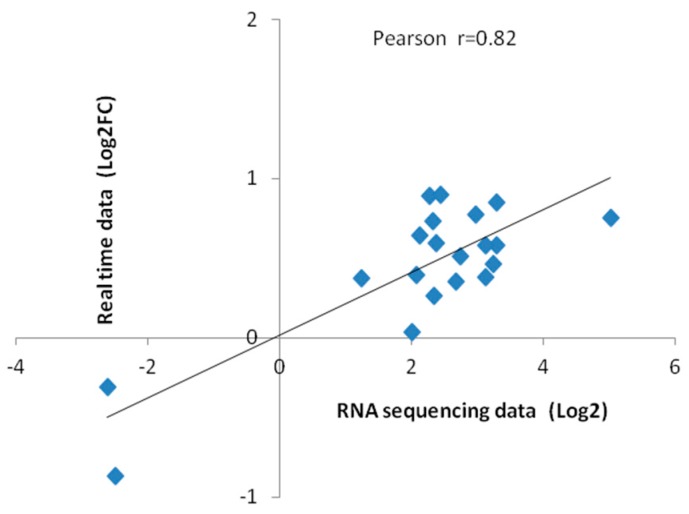
Correlation between sequencing data and quantitative RT-PCR data. Each point represents a value of expression level. Both the X and Y-axes are shown in log_2_ scale. And r indicates the correlation coefficient.

To verify the reliability of sequencing, the RNA samples previously collected for RNA sequencing were used for quantitative real-time PCR. Twenty differential genes were randomly selected for qRT-PCR validation. *GhActin* was selected as the reference gene and the 2^−ΔΔCT^ method was applied to calculate the relative gene expression level. Fluorescence quantitative results and transcriptome sequencing data were as listed in Table 2, which was highly correlated by correlation analysis (Figure 8).

### 2.9. Silencing of GhHMAD5: Phenotype and Expression

**Figure 9:**
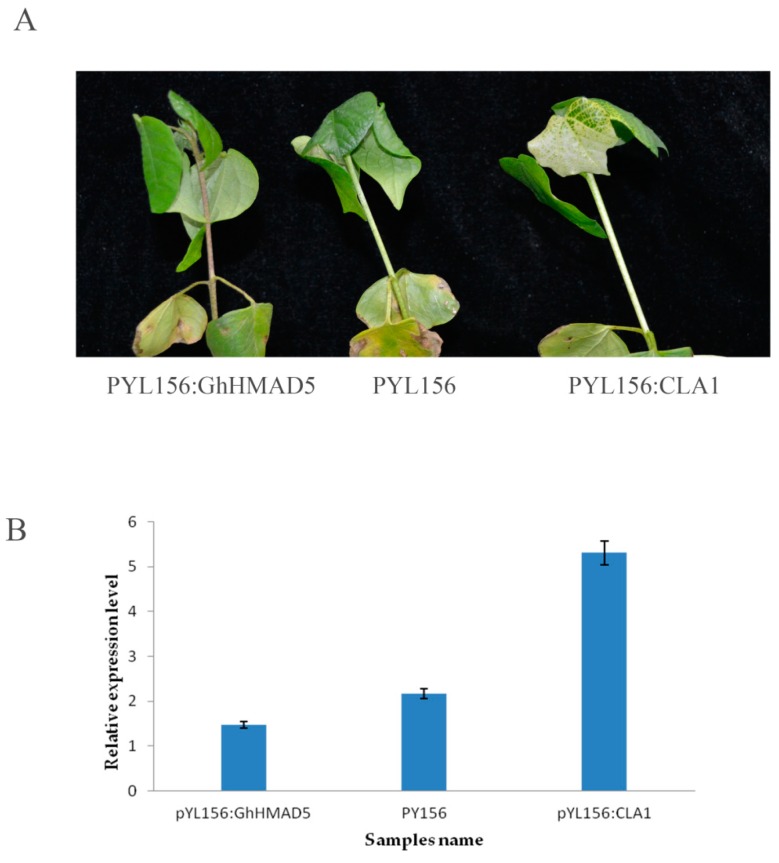
Phenotypic and expression analysis of 4 mM seedlings exposed to Cd stress and the control. (**A**) Phenotypic symptoms of cotton seedlings under 4 mM Cd stress for 9 h. (**B**) The expression analysis of *GhHMAD5* gene of cotton seedlings under 4 mM Cd stress for 9 h.

The cotyledon flattened cotton seedlings were injected with *Agrobacterium tumefaciens*. After albino symptoms appeared in the leaves of positive plants, the infected plants were treated with 4 mM Cd solution. After being exposed to Cd stress for 9 h, the phenotypic changes were obvious. The seedlings, which were soaked in Cd solution, showed the infection, wilted extensively, and the stem darkened and the veins turned brown. The phenotypic symptoms of non-disseminated plants were not obvious (Figure 9A). The expression level of the *GhHMAD5* gene in the plants decreased significantly on exposure to Cd stress (Figure 9B). Furthermore, results showed that after the gene *GhHMAD5* was silenced, the Cd resistance of cotton seedlings decreased.

### 2.10. Overexpression of GhHMAD5 Could Enhance the Cd Tolerance of Arabidopsis Thaliana

**Figure 10:**
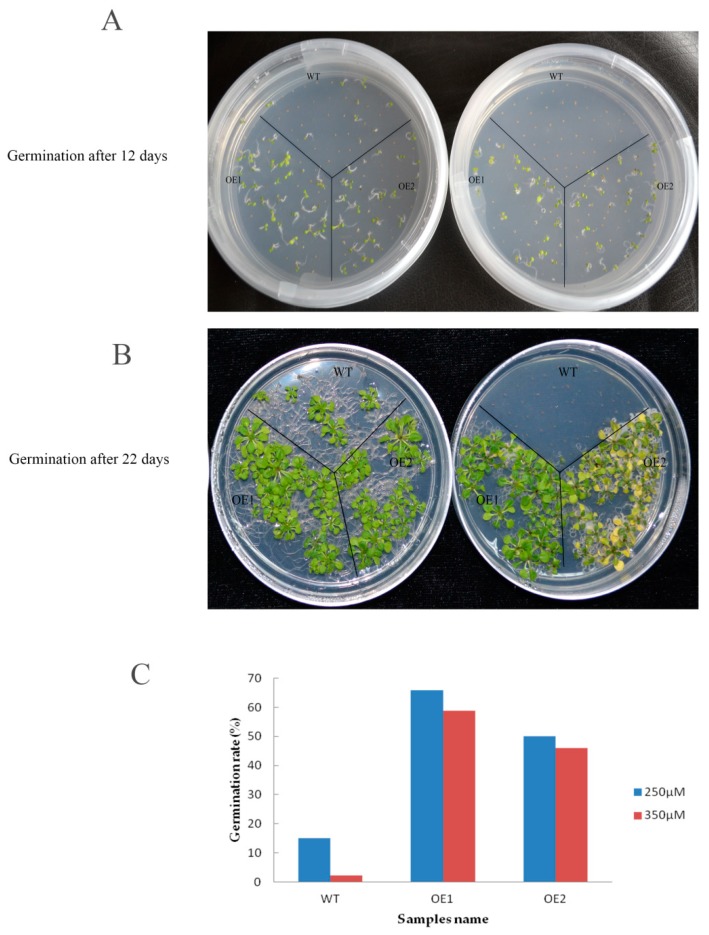
The germination of transgenic *Arabidopsis* and wild type (WT) under Cd stress. (**A**) 12 days of seed germination of transgenic *Arabidopsis thaliana* plants and WT; (**B**) 22 days of seed germination of transgenic *Arabidopsis thaliana* plants and WT. (**C**) The germination rate of transgenic *Arabidopsis thaliana* plants and WT under 250 and 350 μM Cd stress for 22 days. WT represents the wild *Arabidopsis thaliana*. OE1 and OE2 represent the transgenic *Arabidopsis thaliana*.

To examine the function of *GhHMAD5*, the seeds of *GhHMAD5* overexpression plants and WT plants were seeded on 1/2 MS solid medium containing 250 and 350 μM Cd solution for 12 days (Figure 10A) and 22 days (Figure 10B). The viability of WT decreased sharply and significantly with increasing Cd concentration, while the germination of *GhHMAD5* overexpression plants was significantly higher than WT plants. In addition, the germination rate of transgenic plants was not significantly affected by the increase in concentration of Cd stress (Figure 10C), which indicates that overexpression of the *GhHMAD5* gene could improve the germination rate of transgenic *Arabidopsis thaliana*.

**Figure 11:**
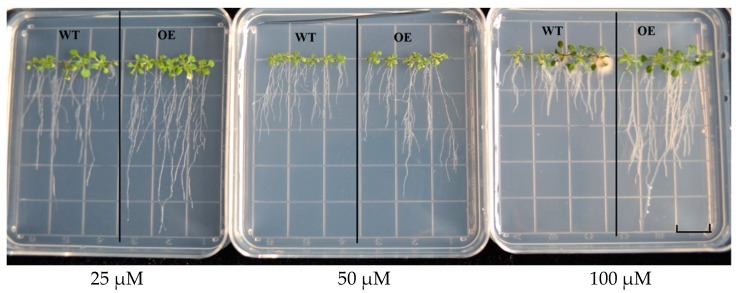
The phenotypic analysis of root length in wild type (WT) and transgenic *Arabidopsis thaliana.* WT represents the wild *Arabidopsis thaliana*, OE represents the transgenic *Arabidopsis thaliana*. Scale bars: 1.4 cm.

Transgenic plants and WT seeds grew on 1/2 MS solid medium containing 25, 50 and 100 μM Cd solution for 12 days (Figure 11). There was no difference in root length between transgenic seedlings and WT plants under 25 μM Cd stress, while under 50 μM and 100 μM Cd stress, the root length of transgenic seedlings was significantly longer than WT plants. These results indicate that overexpression of *GhHMAD5* gene can enhance the Cd tolerance of transgenic *Arabidopsis thaliana*.

## 3. Discussion

Cadmium, one of the common toxic heavy metals that pollute most farmland when exceeding 3.4 mg/kg in the soil, represents a severe threat to plants, animals, and human beings, and is a major problem concerning our health in daily life [39]. Plants suffering from Cd stress showed toxic symptoms, which subsequently resulted in poor quality and diminishing yield [40]. Root tips of broad bean seedlings contaminated with Cd exhibited necrosis and turned dark brown [41]. The morphological indexes of soybean infected by Cd, such as the root length, number of lateral roots, and total volume of the roots decreased as compared to those of the control. Compared with normal plants, the leaf color turned lighter, and the biological yield decreased, and ultimately, the plant died [42]. In this study, we discovered a series of phenotypic symptoms of the cotton root system under Cd stress (Figure 1A). The basal part of the stem lost its water potential and turned black, the petiole of the cotyledon turned black, and the veins became brown, all of which indicate the poisonous effects of Cd stress. However, no significant change in root color and root hair color were observed in cotton under Cd stress, followed by no obvious change in root length and number of roots, which may be related to the time and concentration of Cd stress. In Figure 1B, the Cd content in roots was very high. This indicated that cotton has a strong cadmium accumulation capacity, and cotton roots possess a different cadmium tolerance transport mechanism compared to other crops.

Transcriptome analysis of cotton roots, stems and leaves under Cd stress was carried out in this study. A large number of DEGs were found in roots, followed by leaves and stems. A series of enzymes related to oxidative stress were expressed in cotton under Cd stress, including many emergency proteins, such as heat shock protein and ubiquitin enzyme. The expression of phytochelatin genes was observed in roots, but not in stems and leaves, which shows its tissue specificity for Cd stress. Heavy metal transport proteins played an important role in plant tolerance to Cd stress. It was very interesting that heavy metal transport/detoxification (HMAD) superfamily proteins were found in cotton under Cd stress, which was unknown to previous studies. These proteins contain a heavy-metal-associated domain (HMAD). Some heavy metal transport/detoxification proteins have been shown to be involved in tolerance to toxic metals, such as Pb and Cd [43].

**Figure 12:**
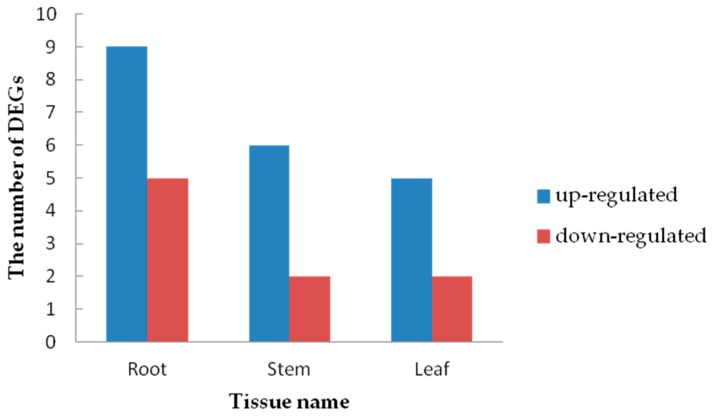
The number of heavy metal transporters/detoxification genes up- and down-regulated in the roots, stems and leaves under Cd Stress.

In this study, 30 DEGs (root: 14 DEGs, stem: 9 DEGs, leaf: 7 DEGs) encoding heavy metal transporters/detoxification superfamily proteins were found (Figure 12). Different tissues contained different HMAD genes (Table S2). These results indicate that the genes belonging to heavy metal transport/detoxification superfamily proteins responded well to Cd stress, and these genes were able to transport and detoxify the heavy metal ions [44]. By silencing the *GhHMAD5* gene in cotton and overexpressing it in *Arabidopsis thaliana*, we found that the *GhHMAD5* gene enhanced Cd resistance, which provided a basis for molecular breeding to remove heavy metal pollution.

Aldolase-type TIM barrel family proteins are divided into two categories based on their functional analysis. One is chlorophyllin, which participates in starch synthesis, and the other is cytoplasmic, involved in the biosynthesis of sucrose [45]. Overexpression of *fructose 1,6-bisphosphate aldolase* (*FBA*) increased the proline content in transgenic plants under salt stress, which enhanced the salt tolerance in tobacco [46]. Gh_D12G1971 was highly expressed in the roots, stems, and leaves of cotton under Cd stress, indicating that HSP protein is related to Cd stress. Cotton could induce the production of an emergency protein kinase in the plant under short-term high Cd stress, which is consistent with previous studies. The function of these two genes needs further investigation.

**Figure 13:**
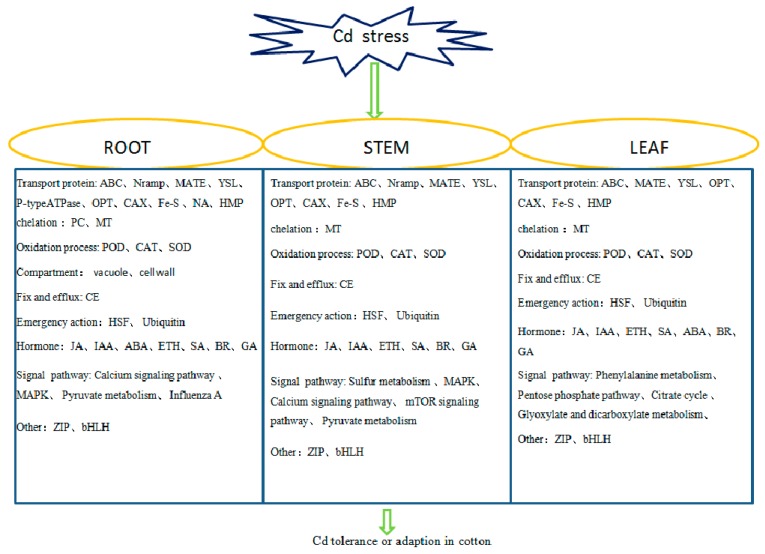
Key factors of Cd tolerance in roots, stems, and leaves of cotton.

**Figure 14:**
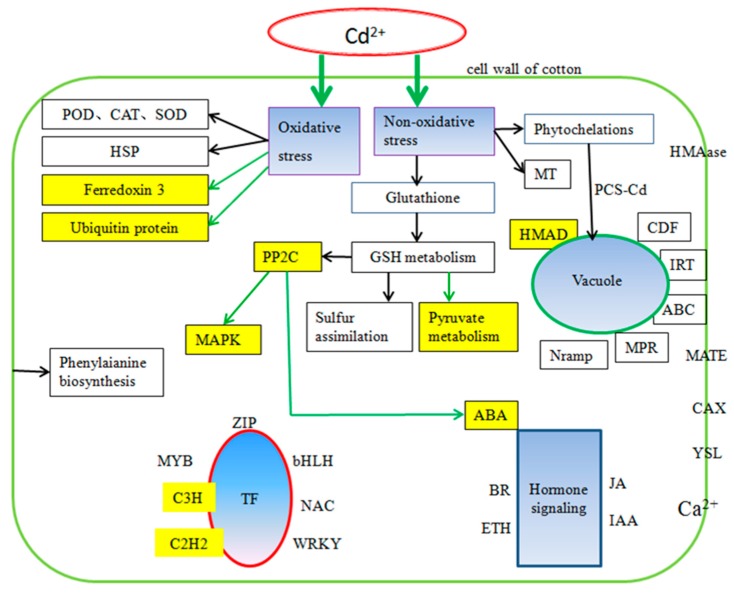
The regulatory network of Cd stress in cotton. The blue fill indicates the main reaction caused by Cd stress. The yellow fill shows a result different from other plants. The yellow background with black characters represents what was previously unknown.

Metabolic pathways of cotton under Cd stress were found to be the same as those reported in other crops, such as carbon metabolism, amino acid biosynthesis, the calcium signaling pathway and the MAPK pathway. Unlike previous reports, 11 DEGs in the roots were enriched in the influenza A pathway (Figure S3), which may indicate a certain correlation between influenza and Cd stress. Cd uptake by plants could lead to DEGs being differentially expressed in the influenza pathway, a necessary process for plants to fight against stress. We found 9 DEGs in cotton which induced pyruvate metabolism (Figure S4), which is the intermediate product playing a key role in the metabolism of sugars, fats and amino acids in plants. Detoxification complexation, thickening of physical barriers and oxidation stress were the main mechanisms in cotton under Cd stress [47]. In this study, we found *GhHMAD5* played an important role in cotton under Cd stress. The *GhHMAD5* gene may be associated with detoxification. According to the DEGs in cotton, we identified the main factors of cotton tolerance to Cd stress (Figure 13). There were complex relationships between PP2C, MAPK and ABA under Cd stress. Ferredoxin 3 and ubiquitin protein also responded to Cd stress in cotton. Above all, we have developed a new prospect for understanding Cd tolerance in cotton (Figure 14), and a new approach for cotton to exploit the Cd tolerance mechanism, which may provide a novel strategy to decode the mechanism of Cd resistance in cotton.

As shown in Figure 14, there were many correlations among the regulatory mechanisms of Cd stress in cotton plants, which coincides with previous studies in other species. The previous study showed that Cd stress caused damage to the antioxidant system of Chinese cabbage, and influenced the expression of genes related to ascorbic acid synthesis to varying degrees [48]. Salicylic acid (SA) was reported to enhance the Cd-tolerance in grapes [49]. A correlation between oxidative stress and ethylene (ETH) signal transduction was investigated under Cd stress [50]. In this study, many hormone-related genes were differentially expressed (Table S3) including SA, ETH, ABA and so on. PP2Cs played a key role in various transduction pathways, such as ABA, pathogeny, stress, and growth, which was consistent with the results of a previous study [51]. The mechanism of Cd tolerance correlates with carbon metabolism, the MAPK signal pathway, and transduction of hormone signals, which implies that Cd stress signals are transduced by hormone networks instead of by a single hormone.

As shown in Table S4, methyltransferase and methyltransferase-related proteins were expressed differentially in cotton under Cd stress, which coincided with previous studies in rice. It was reported that a great number of methyltransferase genes and DNA-methylation-modified genes were expressed differentially in rice under Cd stress [52]. This study suggests DNA methylation is a vital mechanism among complex pathways under Cd stress in plants.

## 4. Materials and Methods

### 4.1. Plant Materials and Cd Stress Treatment

Initially, the experiment was performed on 86 different cotton varieties to check their germination response against several concentrations of Cd stress. Han 242, cotton cultivars (*Gossypium hirsutum* L.), provided by Cotton Research Institute of CAAS, showed the highest germination rate among all varieties under Cd stress. Seeds were washed thoroughly and sown in pots containing well washed, clean, and sterilized sand (121 °C for 8 h). Four seedlings in each pot were cultivated in a 28 °C/14 h light and 25 °C/10 h dark cycle with a light intensity of 150 μmol m^−2^ s^−1^ and 75% relative humidity. After 35 days, cotton plants with three true leaves and one heart-shaped leaf were washed out carefully, and transferred into conical flasks containing 0.5, 1, 2, 4, 6, 8 and 10 mM CdCl_2_ solution to observe various phenotypic changes in a time series of 1, 3, 6, 9, 12, 24 h. Then 4 mM CdCl_2_ over 9 h was chosen as the most appropriate treatment for further study. Seedlings transplanted into ddH_2_O were used as the controls. After exposure for 9 h, leaf, stem and whole root samples were collected. Samples from whole root hair, young stalks and antepenultimate leaves were collected with three replications for each treatment and control to measure the Cd content and the transcriptome analysis.

The wild type *Arabidopsis thaliana* was used for overexpression tests. All *Arabidopsis thaliana* seeds (treated with 70% alcohol for 15 min, and rinsed with double distilled water 6–8 times, before 0.05% agarose was used to suspend the seeds) were cultured in MS medium with 4 °C/24 h dark for 3 days in refrigerator, then transferred into the growth chamber at 20 °C/16 h light and 18 °C/8 h dark cycle for one week. Seedlings were transplanted into soil pots with the environmental conditions of 20 °C/8 h light and 18 °C/16 h dark for one week. After that, growth conditions were replaced by 20 °C/16 h light and 18 °C/8 h dark until their maturation.

### 4.2. Measurement of Relative Cd Content

All samples were oven-dried at 80 °C for 3 days followed by the measurement of Cd content under Cd stress and controls. The concentration of Cd in the filtrate was determined by inductively coupled plasma atomic emission spectroscopy (ICP-P6300) following the standard procedures.

### 4.3. RNA Extraction, cDNA Library Construction, and RNA-Seq

The total RNA was extracted using Trizol reagent (Invitrogen, Carlsbad, CA, USA) following the manufacturer’s procedure. Total RNA quantity and purity were measured with Bioanalyzer 2100 and RNA 6000 Nano LabChip Kit (Agilent, Santa Clara, CA, USA) with RIN number >7, followed by gel extraction with 1% agarose gel electrophoresis. Then approximately 10 µg of total RNA was purified using poly-T oligo-attached magnetic beads cleaved into smaller fragments with fragmentation buffer. Then the cleaved RNA fragments were transcribed to first-strand cDNA fragments using reverse transcriptase and a high concentration of random hexamer primer. The cDNA library was developed by the protocol for the RNA-Seq sample preparation kit (Illumina, San Diego, CA, USA). The average insert size for the paired-end libraries was 150 bp (±50 bp). Paired-end sequencing was performed on an Illumina Hiseq4000 (LC Sciences, San Diego, CA, USA) following the protocols.

### 4.4. Quality Control, and DEG Analysis

Raw data in the fastq format was first processed through in-house perl scripts followed by removal of adaptor and low-quality sequence reads from the data sets. The TopHat package [53] was used to compare the valid dates with the cotton reference genome (*Gossypium hirsutum* L.). These mapped reads were spliced using *Cufflinks* software based on the reference genome sequence. The gene expression levels were calculated using reads per kilobase per million reads (RPKM) [54], which eliminated the influences of gene length and sequencing level during the calculation of gene expression. DEGs were identified using DESeq software [55]. Considering the biological replication, we used the ballgown package in R language to analyze the gene difference after completion of String Tie assembly and quantitation (*p* < 0.05 or q < 0.05) [56]. Fold change ≥ 2 and *p* value < 0.05 were taken as the thresholds to determine whether a gene had differential expression or not.

### 4.5. Gene Ontology and Gene Pathway Enrichment Analysis

To study the DEGs of cotton in response to Cd stress, several bioinformatics tools were employed for annotation, classification and metabolic pathway analysis. The GO enrichment analysis of the DEGs was performed based on agriGO. We identified markedly enriched metabolic pathways or signal transduction pathways in the DEGs based on KEGG [57] and KOBAS [58]. KOBAS software was used for testing the enrichment statistics in the KEGG pathway.

### 4.6. Quantitative Real-Time PCR Analysis

To verify the reliability of the DEGs in RNA-Seq data, we used the same sample as the qRT-PCR analysis. Twenty genes from the DEGs were selected randomly and primers were designed by NCBI (Primer-Blast). The primer sequence of the DEGs and the reference genes are listed in a supplementary table (Table S1). qRT-PCR was performed by using the GeneApplied Biosystems@ 7500 Fast and TransStart Top Green qPCR SuperMix (TransGen Biotech, Beijing, China). Reactions were performed with three technological and biological replications: 0.4 μL of each primer (10 μM), 0.4 μL of passive reference dye, and 10 μL of Top Green qPCR SuperMix at a final volume of 20 μL. The PCR setting was configured as follows: 5 min at 95 °C followed by 40 cycles of amplification at 95 °C for 15 s, then 58 °C for 20 s, and 72 °C for 30 s. The relative fold change of the DEGs was calculated by the 2^−^^∆∆Ct^ [59]. The *GhActin* gene was used as the control, and the correlation analysis between qRT-PCR and RNA-Seq was performed.

### 4.7. Overexpression and Virus Induced Gene Silence (VIGS) Construction of GhHMAD5

The cloning of the gene, named as GhHMA1-t, was done successfully in the early stage by using pEASY-Blunt Kit. Two vectors, pBI 121 vector and pYL 156 were used for overexpression and gene silencing, respectively. The enzyme sites for the pBI 121 vector were *Sac*I and *Sma*I, whereas for pYL 156, they were *EcoR*I and *Xma*I. The linearized gene was connected with its vectors separately and transformed into *E. coli* bacteria by In-Fusion connecting under the heat shock methodology and the results were checked by gel electrophoresis. For further verification and confirmation, samples were sequenced by Sangon Biotech (Shanghai, China) Co., Ltd. The recombinant expression vector plasmids pBI 121: GhHMAD5 and pYL 156: GhHMAD5 were introduced into the *Agrobacterium tumefaciens* strain GV3101 by the freezing and thawing method. Following that, they were stored −80 °C with 50% glycerin to protect bacteria. Overexpression of *GhHMAD5* was carried out in wild type *Arabidopsis thaliana* with pBI 121 vector, while silencing of the gene *GhHMAD5* was accompanied in the cotton cotyledon.

### 4.8. Analysis of Cd Tolerance in Transgenic Arabidopsis Thaliana

Wild-type *Arabidopsis thaliana* was cultivated until florescence, the top inflorescence was cut off for the first time. *Agrobacterium tumefaciens* containing the gene was transferred into 120 mL LB solution with overnight shake until its OD_600_ values reached up to 1.2–1.6. The bacterial solution was centrifuged and sediments were transferred in suspension liquid solution (1/2 MS salt, 5% sucrose, pH = 5.8) and the OD_600_ was set to 0.8. Silwet-L-77 (0.02%) was added into the infection solution uniformly just before its application up to 60 s, while in the second infection time, it was prolonged up to 90 s. The infection plants were wrapped with clean plastic film to maintain humidity. After 16–24 h of dark culture, the infected plants were transferred to previous normal environmental conditions. In order to improve the transformation rate, they were re-infected 5–7 days later. After maturation, the transgenic seeds of the T_0_ generation were obtained.

Cephalosporin (200 mg/L) and kanamycin (50 mg/L) were used to identify the Cd tolerance seedlings from the T_0_ generation seeds. At the 5–7 leaves stage, one leaf was taken as a sample from *Arabidopsis thaliana* plants. DNA extraction was done by the CTAB method for further molecular analysis. Then the T_1_ seeds were obtained. Kanamycin was used to identify the T-DNA insertion line from T_1_ generation seeds. The kanamycin tolerance seedlings isolation rate was 3:1, which proved that the transgenic line was a single T-DNA insertion line. Finally, the transgenic *Arabidopsis thaliana* seeds were obtained.

The seeds of transgenic and wild *Arabidopsis thaliana* were sown on a 1/2 MS solid medium containing 250, 350 μM CdCl_2_. After 12 days and 22 days, the germination rate was observed and analyzed.

Wild type and transgenic *Arabidopsis* seeds were seeded on 1/2 MS solid medium. After 12 days, all seedlings, including wild type and transgenic *Arabidopsis*, were transplanted to a 1/2 MS solid culture plate containing 25, 50 and 100 μM of CdCl_2_ to observe their root development.

### 4.9. The VIGS Analysis of GhHMAD5

Cotton Seeds were washed thoroughly and sown in sand soil pots and the sands were sterilized (121 °C for 8 h). Pots were transferred to incubators with the controlled conditions of 28 °C/14 h light and 25 °C/10 h dark cycle with a light intensity of 150 μmol m^−2^ s^−1^ and 75% relative humidity. After 5 days, a gas-bacilli began to sprout (in cotton seedlings two cotyledons flattened first and then grew into leaves). The saved *Agrobacterium* solution was shaken in 60 mL LB liquid medium, until its OD_600_ value reached 1.5, which was followed by centrifugation. *Agrobacterium* was dispersed in suspension solution (10 mM MgCl_2_ + 10 mM MES + 200 μM acetosyingone). Suspension solutions of pYL 156: GhHMAD5, pYL 156: GhCLA1 and pYL 156 were mixed with equal volumes of assistant bacteria auxiliary carrier pYL 192 prior to injection in cotyledons. Cotyledons were injured on their back with a sterilized needle tab and the bacterial suspension solution was injected in this hole for microbial proliferation on the whole cotyledon. Cotton plants were transferred to an incubator in darkness for 24 h at 23 °C. Later on, it was replaced by normal environmental conditions (28 °C/14 h light, 25 °C/10 h dark). After being subjected to blanching, plants were treated with 4 mM Cd stress which showed the symptoms of positive seedlings very clearly as compared to negative ones. Relative expressions were measured at this stage.

## 5. Conclusions

In this study, 11,503 DEGs were discovered under Cd stress in cotton with RNA-Seq analysis. The *GhHMAD5* gene was cloned and identified as enhancing the resistance to Cd stress. A novel regulation network of Cd stress was constructed, including complex pathways, in cotton. This study suggests a preliminary understanding of Cd tolerance mechanisms in upland cotton, which implies a potential use of cotton is to remediate Cd-polluted soil.

## Figures and Tables

**Table 1:** Overview of the transcriptome sequencing of upland cotton. R_T1: Root Cd treatment 1, R_T2: Root Cd treatment 2, R_T3: Root Cd treatment 3, R_C1: Root water control 1, R_C2: Root water control 2, R_C3: Root water control 3, S_T1: Stem Cd treatment 1, S_T2: Stem Cd treatment 2, S_T3: Stem Cd treatment 3, S_C1: Stem water control 1, S_C2: Stem water control 2, S_C3: Stem water control 3, L_T1: Leaf Cd treatment 1, L_T2: Leaf Cd treatment 2, L_T3: Leaf Cd treatment 3, L_C1: Leaf water control 1, L_C2: Leaf water control 2, L_C3: Leaf water control 3.

Sample	Number of Raw Reads	Number of Valid Reads	Q20 Percentage (%)	Q30 Percentage (%)	GC Content (%)
R_T1	62,264,174	61,471,556	98.04	91.00	45
R_T2	55,526,318	54,811,464	97.93	91.32	45
R_T3	57,133,152	56,462,772	98.27	92.14	45
R_C1	55,124,734	54,473,502	97.93	90.83	45
R_C2	65,102,420	64,390,908	98.45	92.15	44
R_C3	52,774,982	52,144,004	97.78	90.97	45
S_T1	54,098,272	53,474,704	99.35	95.05	44
S_T2	43,663,718	43,152,570	99.19	95.13	45
S_T3	43,207,572	42,631,528	99.43	96.20	44
S_C1	57,188,916	56,544,630	98.99	94.40	45
S_C2	42,417,384	41,057,536	98.01	89.91	44
S_C3	50,332,770	48,940,728	98.28	90.06	45
L_T1	42,697,218	41,184,508	98.22	89.03	44
L_T2	45,204,982	44,593,254	98.25	89.42	45
L_T3	48,167,202	47,153,586	98.30	90.14	44
L_C1	63,540,928	62,794,552	99.67	96.60	44
L_C2	39,136,086	38,791,254	99.66	95.22	44
L_C3	46,343,822	45,925,388	99.74	95.38	44

**Table 2:** The DEGs validated by qRT-PCR.

Gene ID	RNA-Seq (Log_2_FC)	qRT-PCR (FC)	Description
Gh_A01G1234	3.24	2.89	PLANT CADMIUM RESISTANCE 2
Gh_D11G2939	3.12	2.42	metallothionein 3
Gh_D06G0421	2.97	5.86	NRAMP metal ion transporter 6
Gh_D04G1180	2.68	2.24	Fes1A
Gh_A11G2566	2.37	3.90	metallothionein 3
Gh_A04G0265	2.33	5.37	metallothionein 3
Gh_D08G1950	1.25	2.37	heavy metal atpase 5
Gh_A04G0713	2.12	4.38	Fes1A
Gh_A08G2485	5.03	5.67	AUX/IAA transcriptional regulator family protein
Gh_D07G2124	2.28	7.62	AUX/IAA transcriptional regulator family protein
Gh_D08G2126	2.45	7.96	Heavy metal transport/detoxification superfamily protein
Gh_D04G0262	3.29	3.84	Auxin-responsive GH3 family protein
Gh_D04G0260	3.29	7.16	Auxin-responsive GH3 family protein
Gh_A01G2049	3.13	3.80	Arabidopsis thaliana gibberellin 2-oxidase 1
Gh_A07G1285	−2.49	0.13	Heavy metal transport/detoxification superfamily protein
Gh_D07G1640	2.34	1.80	Heavy metal transport/detoxification superfamily protein
Gh_D10G0078	−2.61	0.49	Heavy metal transport/detoxification superfamily protein
Gh_D13G1609	2.74	3.26	Arabidopsis thaliana gibberellin 2-oxidase 1
Gh_D09G1816	2.01	1.08	Heavy metal transport/detoxification superfamily protein
Gh_A05G0151	2.08	2.43	Heavy metal transport/detoxification superfamily protein

## Supplementary Materials

The following are available online at https://www.mdpi.com/1422-0067/20/6/1479/s1.
