# Supplementary material for: Transcriptome Analysis Reveals Cotton (Gossypium hirsutum) Genes That Are Differentially Expressed in Cadmium Stress Tolerance
Source: Int J Mol Sci. 2019 Mar 24;20(6):1479. doi: 10.3390/ijms20061479 (PMC6470502; doi:10.3390/ijms20061479)
Supplement: Supplementary file 1 [file ijms-20-01479-s001.pdf]

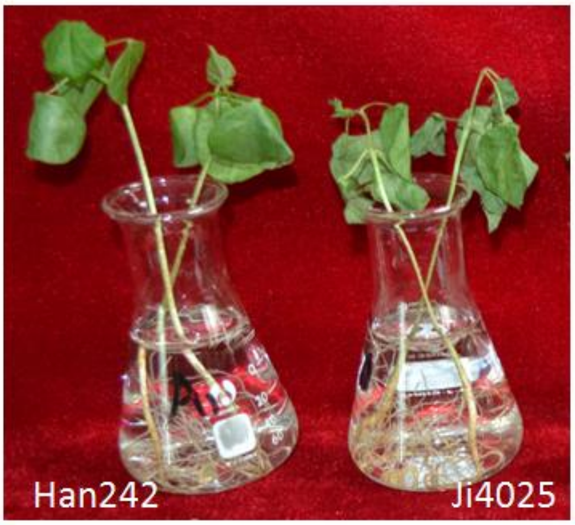

Figure 1. Phenotypes of different cotton varieties under cadmium stress for 4mM Cd.

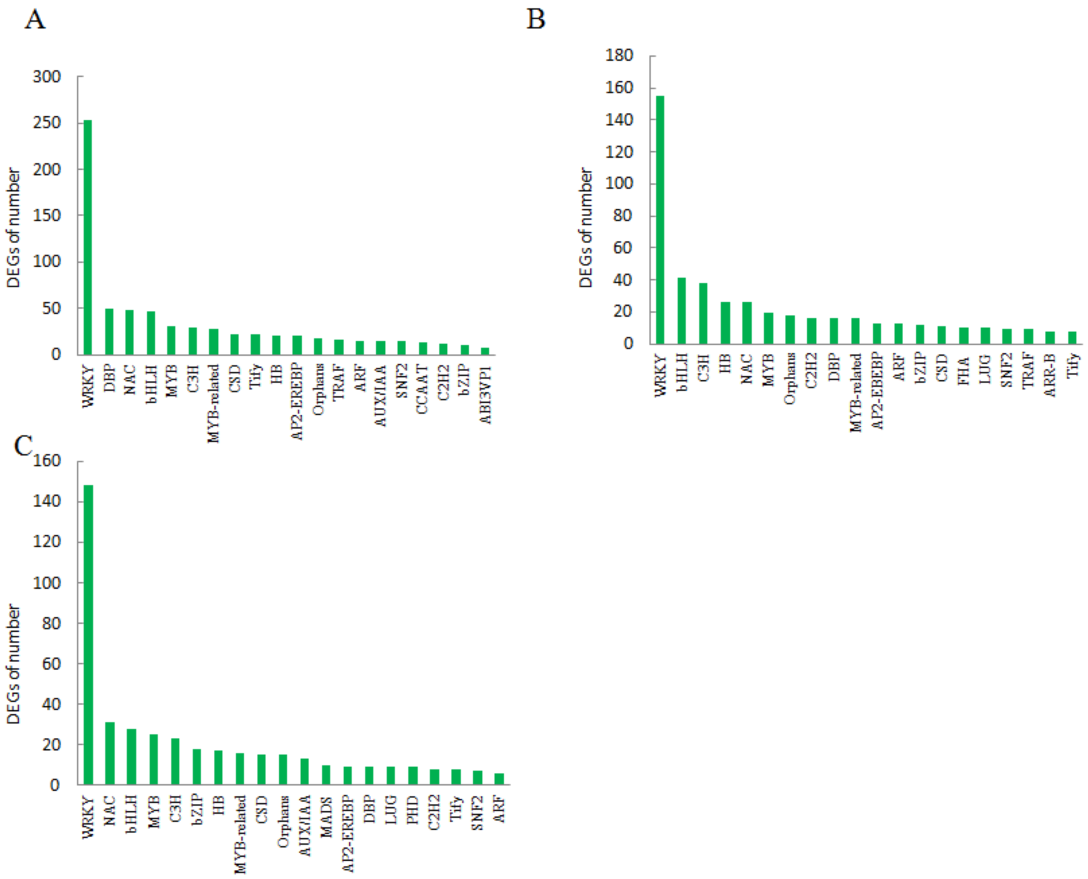

Figure 2. Expression of transcription factors in cotton roots, stems, and leaves under cadmium Stress.

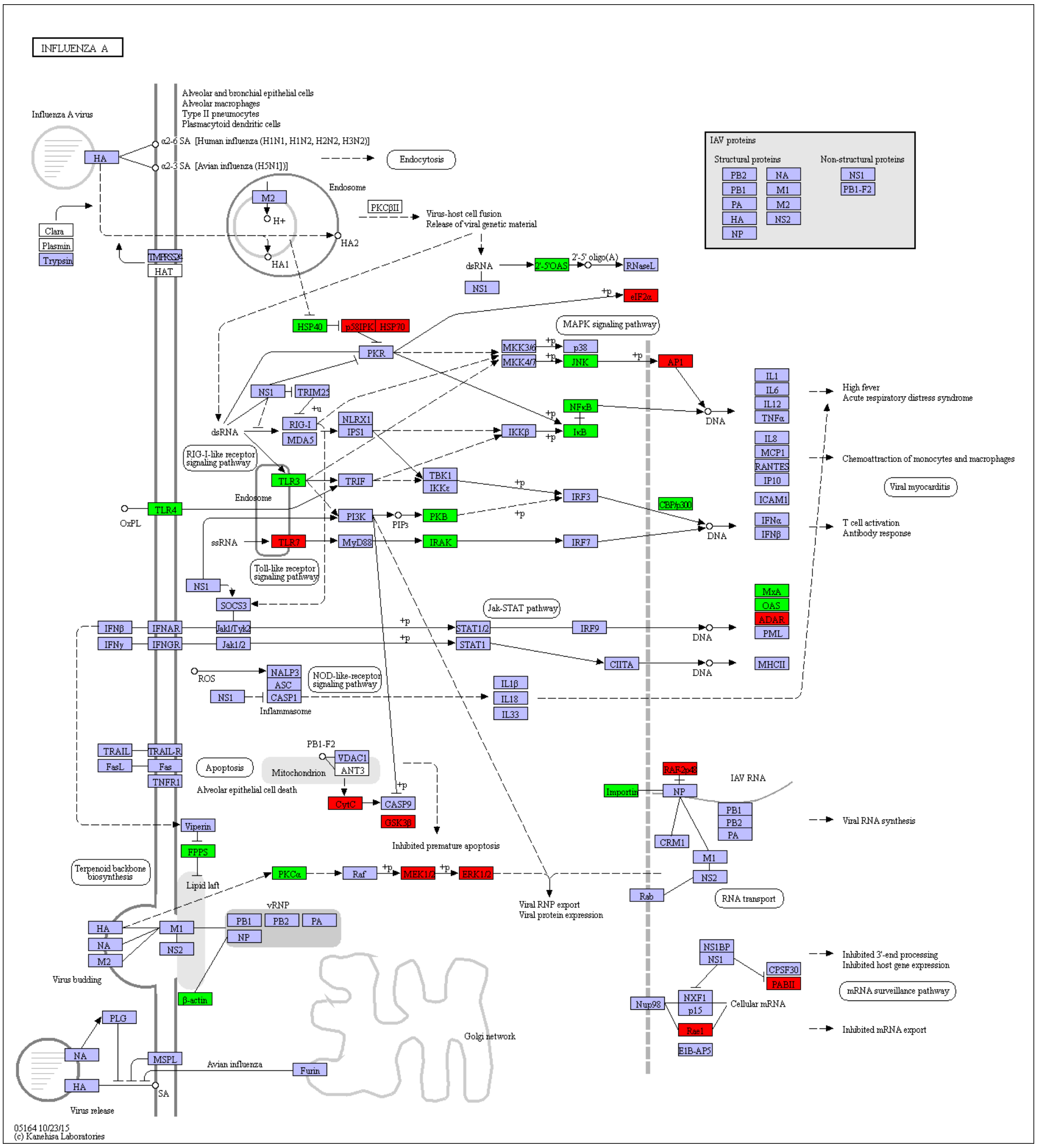

Figure 3. DEGs involved in Influenza A under Cd stress in cotton. Red and green squares indicate up- and down-regulated genes, respectively, and gray squares indicate genes whose expression did not change significantly.

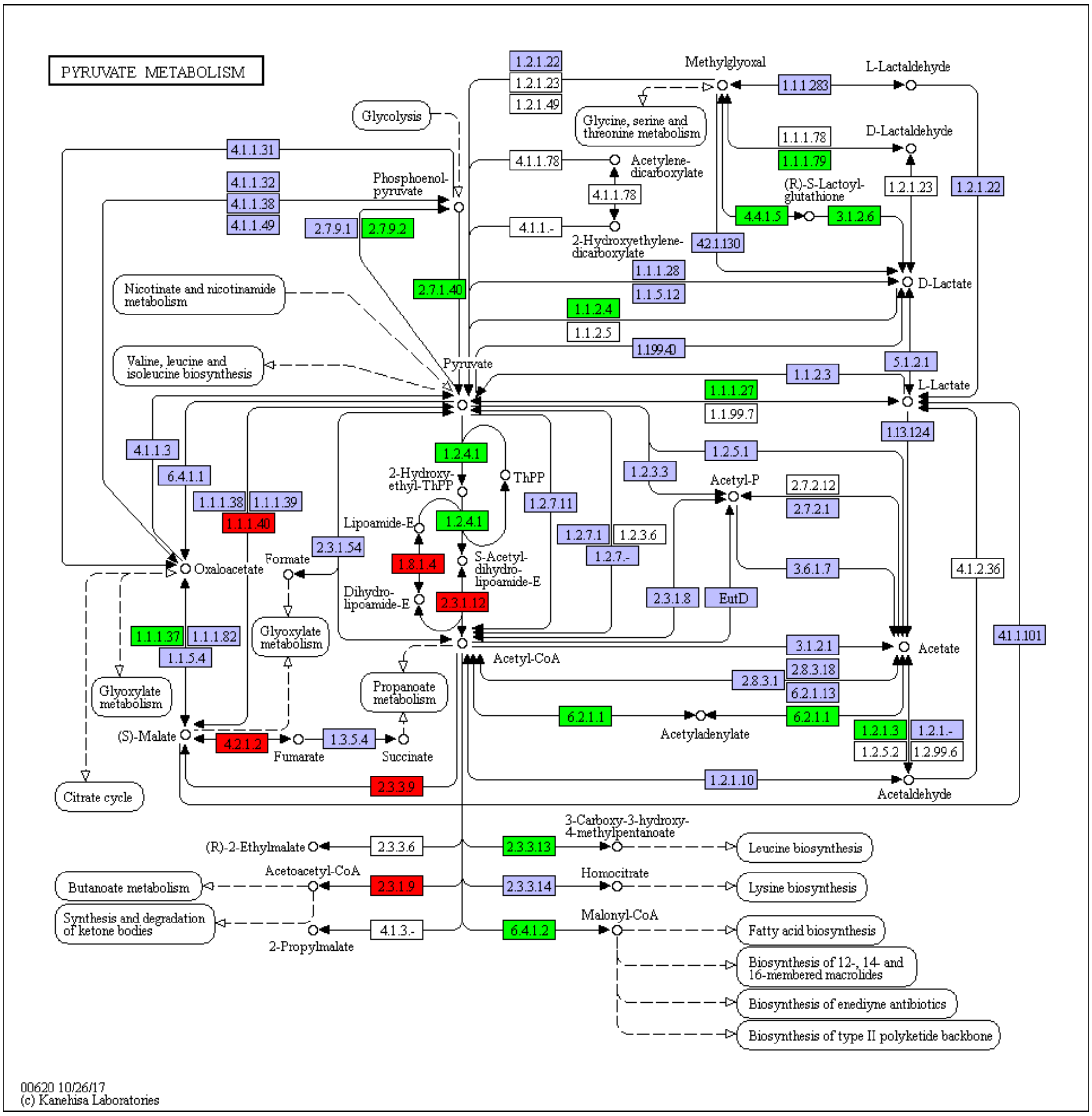

Figure 4. Pyruvate metabolism pathways. Red and green squares indicate up- and down-regulated genes, respectively, and gray squares indicate genes whose expression did not change significantly.

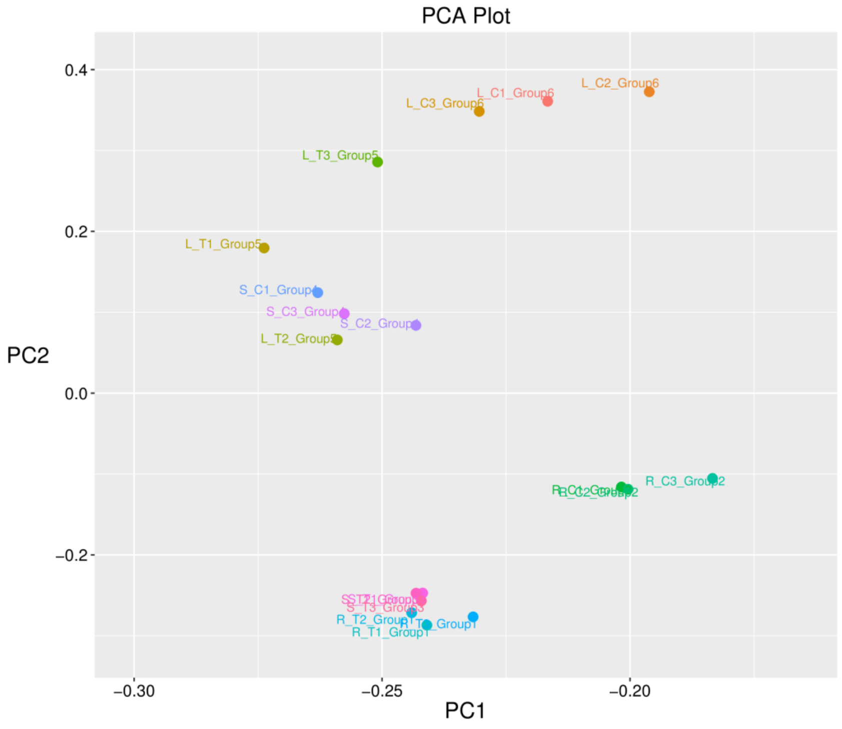

Figure 5. PCA analysis between three samples.

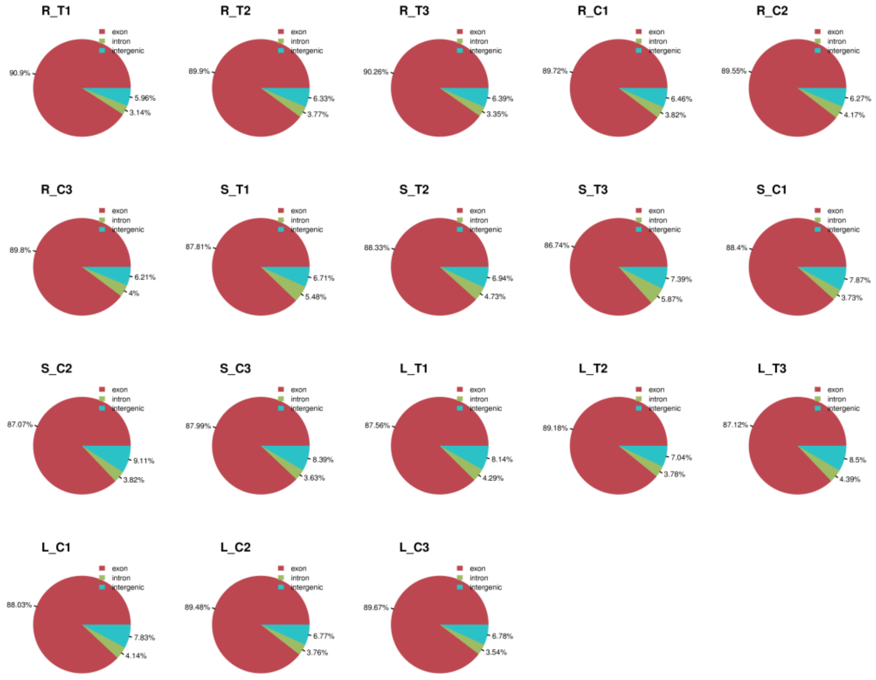

Figure 6. Purified and valid reads in each sample were mapped to the G.hirsutum genome.

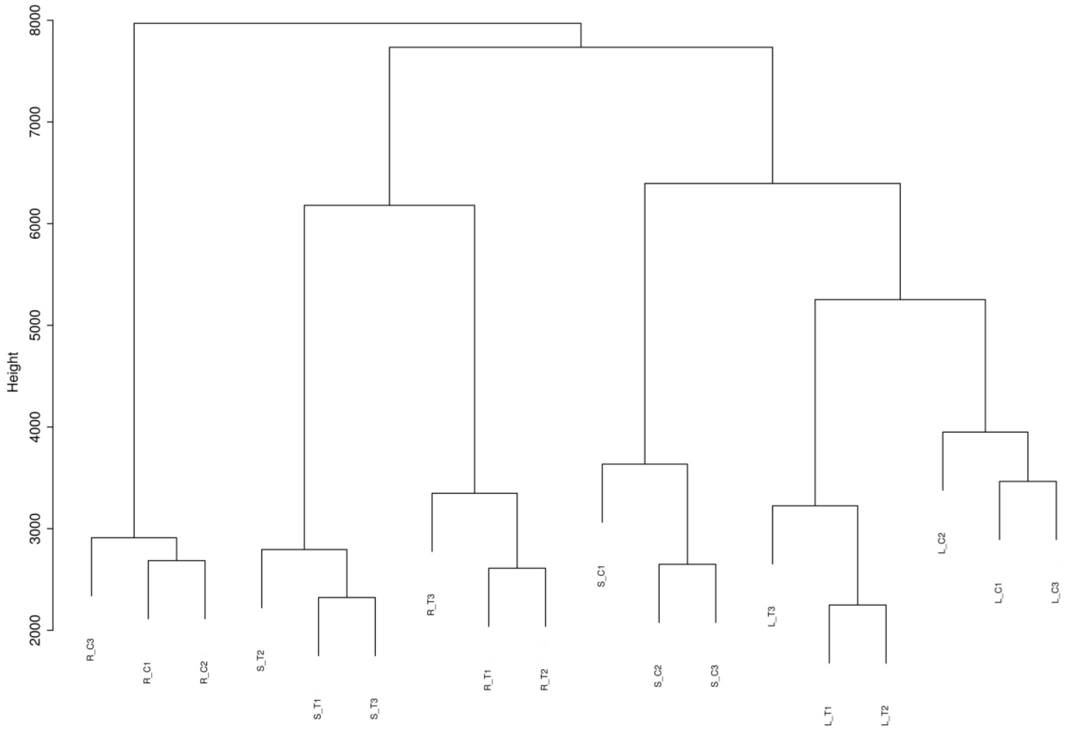

Figure 7. Samples clustering to detect outliers. R_T1: Root Cd treatment 1, R_T2: Root Cd treatment 2, R_T3: Root Cd treatment 3, R_C1: Root water control 1, R_C2: Root water control 2, R_C3: Root water control 3, S_T1: Stem Cd treatment 1, S_T2: Stem Cd treatment 2, S_T3: Stem Cd treatment 3, S_C1: Stem water control 1, S_C2: Stem water control 2, S_C3: Stem water control 3, L_T1: Leaf Cd treatment 1, L_T2: Leaf Cd treatment 2, L_T3: Leaf Cd treatment 3, L_C1: Leaf water control 1, L_C2: Leaf water control 2, L_C3: Leaf water control 3.

Table 1. Gene name, Gene annotation, and primers used for qRT-PCR validation.

Gene name	Primers	Gene	Primers
**Gh_A01G1234**	F: CCCCACGAACCAATCAACCT	**Gh_D04G0262**	F:CACAAGCCCCATTGAGACCA
	R: GCGGTTGCGTGTAATTTTGC		R:CGTTGACAACCTCGTCTCGT
**Gh_D11G2939**	F: GCCAATGCACGAAGAAGGGA	**Gh_D04G0260**	F:ACCCATTACAGGCTCTGGGA
	R: TTGCACTTGCCATCGTTCTC		R:CCAAAGTGCTTCATCGCAGG
**Gh_D06G0421**	F: TGATTGGGACAGCCTTTGGA	**Gh_A01G2049**	F:CCATTTCCGTGTTGGAATCCG
	R: CCCATATCTCTGCAGGCCAA		R:TCAACCCAACCAACATCACCT
**Gh_D04G1180**	F: GGGCTCCCTCGTGTAATGTT	**Gh_A07G1285**	F: CAAAACTGTGGCTGGGTTGA
	R: CAACGTCAAGGAGACCACGA		R:CTGTCAACTGCTTTGCCTTCA
**Gh_A11G2566**	F:ACGAAGGGAAACAGCAACAC	**Gh_D07G1640**	F:GTAGTCGGTTTCTCAGGGGTG
	R: TTGCACTTGCCATCGTTCTC		R:GCTCTTCCTTAGCCGACTTGT
**Gh_A04G0265**	F: TGACAAGAGCCAGTGTGTGAA	**Gh_D10G0078**	F: CCATCACGGTGGTATGCTGT
	R: TTGCACTTGCCATCGTTCTC		R: GTCGGGTTTCTTCTCCGGTT
**Gh_D08G1950**	F: TCGTTCAAGTCTGCCGGATT	**Gh_D13G1609**	F:ATGGACTTCATTTCCCGGCT
	R: TCTGTGCTTCTTCGGTTGCT		R:AGGCTTGGGTTGTCCTGTTT
**Gh_A04G0713**	F: GGGCTCCCTCGTGTAATGTT	**Gh_D09G1816**	F:TTTCCGGTCTCTCCGGTGTT
	R: CAAGCTCAAGGAGACCACGA		R:GCCGGTCCAACCGTTAAAATC
**Gh_A08G2485**	F:GGTGACTGGATGCTTGTTGGA	**Gh_A05G0151**	F:GATGTTGTGGCTGGCAATCAG
	R:TCATACACAGCCCAACCCTC		R:GAGGTCACTCCATCCATCTTTG
**Gh_D07G2124**	F:GTTGAAGGCGGGACAGGAAT	**Ghaction**	F: TGACGGAGAATTAGGGTTCGA
	R:AAAGCCAAGAGGAGTTGAGGG		R:CCGTGTCAGGATTGGGTAATTT
**Gh_D08G2126**	F: GCAAGATGGAAGGGAACCGA		

R: GTGCGGATACGGGTTTCCAT

Table 2. Expression of heavy metal transport/detoxification superfamily proteins under Cadmium Stress in cotton.

tissue	Gene name	FC	regulation
Root			
	Gh_A07G1285	0.18	down
	Gh_A11G2427	0.38	down
	Gh_D07G1640	0.20	down
	Gh_D10G0078	0.16	down
	Gh_D12G1886	0.29	down
	Gh_A08G1780	3.88	up
	Gh_A12G2298	2.59	up
	Gh_D04G1066	3.77	up
	Gh_D07G1023	2.96	up
	Gh_D08G2126	5.48	up
	Gh_D09G1816	4.04	up
	Gh_D11G1515	2.85	up
	Gh_D12G1507	2.82	up
	Gh_A05G0151	4.24	Up
Stem			
	Gh_A06G1738	0.04	Down
	Gh_A12G0960	56.93	up
	Gh_A12G1384	0.08	down
	Gh_D07G2221	11.13	up
	Gh_D08G2126	6.13	up
	Gh_D11G2744	8.05	up
	Gh_D12G1072	29.61	up
	Gh_A05G0151	5.16	up
Leaf			
	Gh_A06G1738	5.20	up
	Gh_A07G1285	5.03	up
	Gh_A09G1374	2.63	up
	Gh_A11G1367	0.24	down
	Gh_D04G1066	19.33	up

	Gh_D07G2221	0.14	down
	Gh_D09G1375	2.69	up

Table 3. The expression of hormone protein under cadmium stress in cotton.

gene	Root	Stem	Leaf
**IAA**	28	15	17
**JA**	24	9	7
**BR**	9	4	5
**ETH**	12	3	16
**ABA**	5	3	4

Table 4. The expression of methyltransferase in cotton by cadmium stress.

transcript_id	Description	FC
**Gh_D11G0656**	*methylesterase 11*	2.49
**Gh_D12G0234**	*methylesterase 3*	2.37
**Gh_D11G3357**	*Methyltransferase-related protein*	2.30
**Gh_A10G0592**	*methylesterase 12*	2.16
**Gh_D11G0488**	*methylesterase 10*	0.43
**Gh_D11G0487**	*methylesterase 10*	0.31
**Gh_A05G2377**	*methylesterase 17*	0.30
**Gh_A11G0421**	*methylesterase 10*	0.25

Table 5. Statistics of functional genes with Cufflinks.

Genes	Root	Stem	Leaf	Total
All gene	43842	45435	45885	135162
Annotation gene	38130	39462	39785	117377
Novel gene	5712	5973	6100	17785
